# The Effectiveness of Surgical Methods for Trismus Release at Least 6 Months After Head and Neck Cancer Treatment: Systematic Review

**DOI:** 10.3389/froh.2021.810288

**Published:** 2022-01-21

**Authors:** Maximiliaan Smeets, Tomas-Marijn Croonenborghs, Jeroen Van Dessel, Constantinus Politis, Reinhilde Jacobs, Michel Bila

**Affiliations:** ^1^OMFS IMPATH Research Group, Department of Imaging and Pathology, Faculty of Medicine, KU Leuven, Leuven, Belgium; ^2^Department of Oral and Maxillofacial Surgery, University Hospitals Leuven, Leuven, Belgium; ^3^Department of Dental Medicine, Karolinska Institute, Stockholm, Sweden

**Keywords:** trismus, coronoidectomy, myotomy, free flap, oral cancer, trismus release

## Abstract

**Background:**

The objective of this systematic review was to identify the different surgical treatment modalities of severe trismus after head and neck squamous cell cancer treatment.

**Methods:**

An electronic literature database search was conducted in Medline, Embase, Cochrane, Web of Science, and OpenGrey to determine articles published up to September 2021. Two observers independently assessed the identified papers for eligibility according to PRISMA guidelines. The inclusion criteria were trismus after head and neck squamous cell cancer with consecutive treatment, detailed description of the surgical procedure for trismus release, description of the initial treatment, at least 6 months between initial cancer treatment and trismus release surgery, a minimal follow-up (FU) of 6 months, and availability of full text. The quality was evaluated using the Newcastle-Ottawa scale. A subanalysis of the maximal mouth opening (MMO) was performed using a mixed-effect model.

**Results:**

A total of 8,607 unique articles were screened for eligibility, 69 full texts were reviewed, and 3 studies, with a total of 46 cases, were selected based on the predetermined inclusion and exclusion criteria. Three treatment strategies were identified for trismus release (1) free flap reconstruction (FFR), (2) coronoidectomy (CN), and (3) myotomy (MT). There was a clear improvement for all treatment modalities. A quantitative analysis showed a beneficial effect of CN (mean 24.02 ± 15.02 mm) in comparison with FFR (mean 19.88 ± 13.97 mm) and MT (mean 18.38 ± 13.22 mm) (*P* < 0.01^*^). An increased gain in MMO after trismus release was found if no primary resection was performed (*P* = 0.014^*^). Two studies included in the analysis had an intermediate risk of bias and one had a low risk of bias.

**Conclusion:**

Currently available reports suggest a low threshold for performing a CN compared with FFR and MT. There is a need for high-quality randomized controlled trials with carefully selected and standardized outcome measures.

## Introduction

Trismus is one of the most evident complications secondary to head and neck squamous cell cancer (HNSCC) treatment, with severe impact on the quality of life [1–3]. The prevalence of trismus after HNSCC treatment varies widely with ranges from 5 to 41.5% [4–12]. The degree of limitation of the maximal mouth opening (MMO) is typically most evident 6 months after treatment [6]. Predictive factors for trismus in HNSCC are still arguable, but despite newer radiation modalities, radiotherapy appears to remain a major contributor to limited MMO [13, 14].

Most patients are treated with conservative tools and instructions to prevent severe trismus. In this context, the early start of exercise therapy is crucial [15–17]. Scherpenhuizen et al. [16] already stated the absolute benefit of exercise therapy over no exercise at all. Multiple tools are currently available for stretching, but a systematic review by Kamstra et al. [15] could not define a preferred exercise therapy. Besides conservative therapy, the role of pentoxifylline is unclear as only one pilot study has covered the effect of pentoxifylline on the mouth opening [18].

In some cases, conservative therapies remain inadequate to reach a sufficient MMO for most essential daily life activities. In cases of intraoral soft-tissue scar tissue caused by reconstructions or radiotherapy, surgical release may be considered. Surgical interventions are based on just one or a combination of different release strategies, namely a myotomy (MT) of the masticatory muscles, a coronoidectomy (CN) and resection of fibrous scar tissue followed with a free flap reconstruction (FFR). No clear therapeutic flowchart for surgical release of trismus is available despite the high prevalence and impact on the quality of life of trismus secondary to the different treatment modalities of HNSCC.

The aim of this systematic review is to identify the surgical methods to improve mouth opening minimally 6 months after HNSCC treatment and to compare their effectiveness on the increase in MMO after surgery.

## Materials and Methods

### Eligibility Criteria

The inclusion criteria were trismus after HNSCC with consecutive treatment, detailed description of the surgical procedure for trismus release, description of the initial treatment, at least 6 months between initial cancer treatment and trismus release surgery, a minimal follow-up (FU) of 6 months, and availability of full text in Dutch, French, English or German. Literature reviews, systemic reviews, histological and animal studies, case reports, and case series with <6 patients were not included in the study selection due to wrong study design but were used as potential sources to find relevant missing articles in the search. This was performed by careful analysis of all referred references in these manuscripts. The study selection was done in two stages, first by screening titles and abstracts, and then by reading the full text article meeting the inclusion criteria. At the end of each stage, a consensus was sought for disagreements.

### Information Sources and Search Strategy

A search strategy was developed for Medline, Embase, Cochrane, Web of Science, and OpenGrey for studies published up to September 2021 (Supplementary Material). Consequently, a thorough manual search was conducted.

### Selection Process

Two reviewers (MS and TMC) independently assessed titles, abstracts, and full text articles following specific eligibility criteria. All references were collected, and duplicates were removed in Covidence systematic review software (Veritas Health Innovation, Melbourne, Australia). The references of the studies that were included for eligibility screening were all carefully analyzed for any additional manuscripts that were not yet detected via the primary search strategy.

### Data Collection Process

Two authors (MS and TMC) independently extracted data from the selected articles.

### Data Items

The following parameters were extracted from each included study: name of first author, year of publication, study design, number of participants, gender, mean age, age range, mean FU time, FU range, surgical intervention, MMO at least 6 months after surgical release. In case of combined or missing parameters, the corresponding authors of the manuscript were contacted by email to request the raw data.

### Study Risk and Reporting of Bias Assessment

Assessment of the quality was achieved with the Newcastle-Ottawa quality assessment scale [19]. This scoring system requires a grading on several domains: possible biases of selection, comparability, and exposure. Scores ranged from 0 (a very biased article) to 9 (bias very unlikely) (Table 1). Studies have a low risk of bias if the score is 7–9, intermediate risk if 4–6, and high risk when the score is below 4. The scores were given by three authors (MS, JVD and TMC) and the mean score was used.

**Table 1:** Quality assessment according to the Newcastle-Ottawa quality assessment scale.

		**References**		
		**Bhrany et al.** ** [20] **	**de Pablo et al.** ** [21] **	**Mardini et al.** ** [22]**
**Item**				
**Selection**	Representativeness of the exposed cohort.	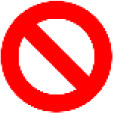	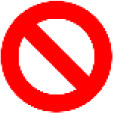	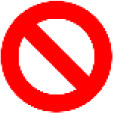
	Selection of nonexposed cohort.	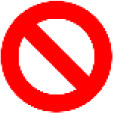		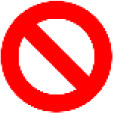
	Ascertainment of exposure.			
	Demonstration that outcome of interest was not present at start of the study.			
**Comparability**	Study controls for other variables.	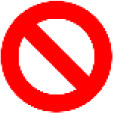 	 	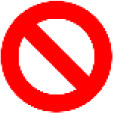 
**Outcome**	Assessment of outcome.			
	Degree of FU was long enough for outcomes to occur.			
	Adequacy of FU of cohorts.			
**SCORE (/9)**		6	8	6

*
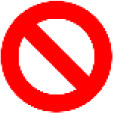
, no agreement; 

, agreement*.

### Synthesis Methods

Data was collected from the articles that met the selection criteria. The effect of the primary therapy on the reversibility of the MMO after trismus release was evaluated via a logistic regression. More specific the use of osteocutaneous and fasciocutaneous flaps, the administration of radiotherapy (yes/no), and the performance of a primary resection (yes/no). Differences were evaluated among 3 possible interventions (1) CN; (2) MT; (3) FFR. The mean MMO was evaluated pre-, peri- and postoperative at the end of FU.

### Protocol and Registration

This systematic review were performed in accordance with a predefined protocol registered in PROSPERO (CRD42020158770). The Preferred Reporting Items for Systematic Reviews and Meta-Analysis (PRISMA) guidelines were followed [23].

### PICO Question

The review was designed based on the following PICOS criteria (population, intervention, comparison, outcome, studies): (P) limited MMO secondary to HNSCC treatment (radiotherapy, surgery, chemotherapy, and/or check-point inhibition therapy), (I) surgical release, (C) different surgical techniques, (O) mean MMO pre-, peri- and postoperative at the end of FU, and (S) all studies except literature reviews, systemic reviews, histological and animal studies, case reports, and case series with <6 patients.

### Selection Process of Studies

Two reviewers (MS and TMC) independently assessed titles, abstracts, and full text articles following specific eligibility criteria. The inclusion criteria were trismus after HNSCC with consecutive treatment, detailed description of the surgical procedure for trismus release, description of the initial treatment, at least 6 months between initial cancer treatment and trismus release surgery, a minimal follow-up (FU) of 6 months, and availability of full text in Dutch, French, English or German. Literature reviews, systemic reviews, histological and animal studies, case reports, and case series with <6 patients were not included in the study selection due to wrong study design but were used as potential sources to find relevant missing articles in the search. This was performed by careful analysis of all referred references in these manuscripts. The study selection was done in two stages, first by screening titles and abstracts, and then by reading the full text article meeting the inclusion criteria. At the end of each stage, a consensus was sought for disagreements.

### Synthesis Methods and Statistical Analysis

A general linear mixed-effects model was applied to examine the influence of the different treatment protocols and time points on the MMO. Bonferroni-corrected *post-hoc t*-tests were used to examine significant main and interaction effects.

The statistical analysis was conducted in IBM SPSS statistical software (Version 22.0, IBM, New York, USA). The significance level α was set for all statistical tests at 0.05.

## Results

### Study Selection

A total of 13,616 articles were identified, and after screening for duplicates, 8,607 unique titles remained. Title and abstract selection resulted in 69 relevant articles for eligibility assessment. After assessment of the full text, 3 papers remained for qualitative synthesis.

**Figure 1:**
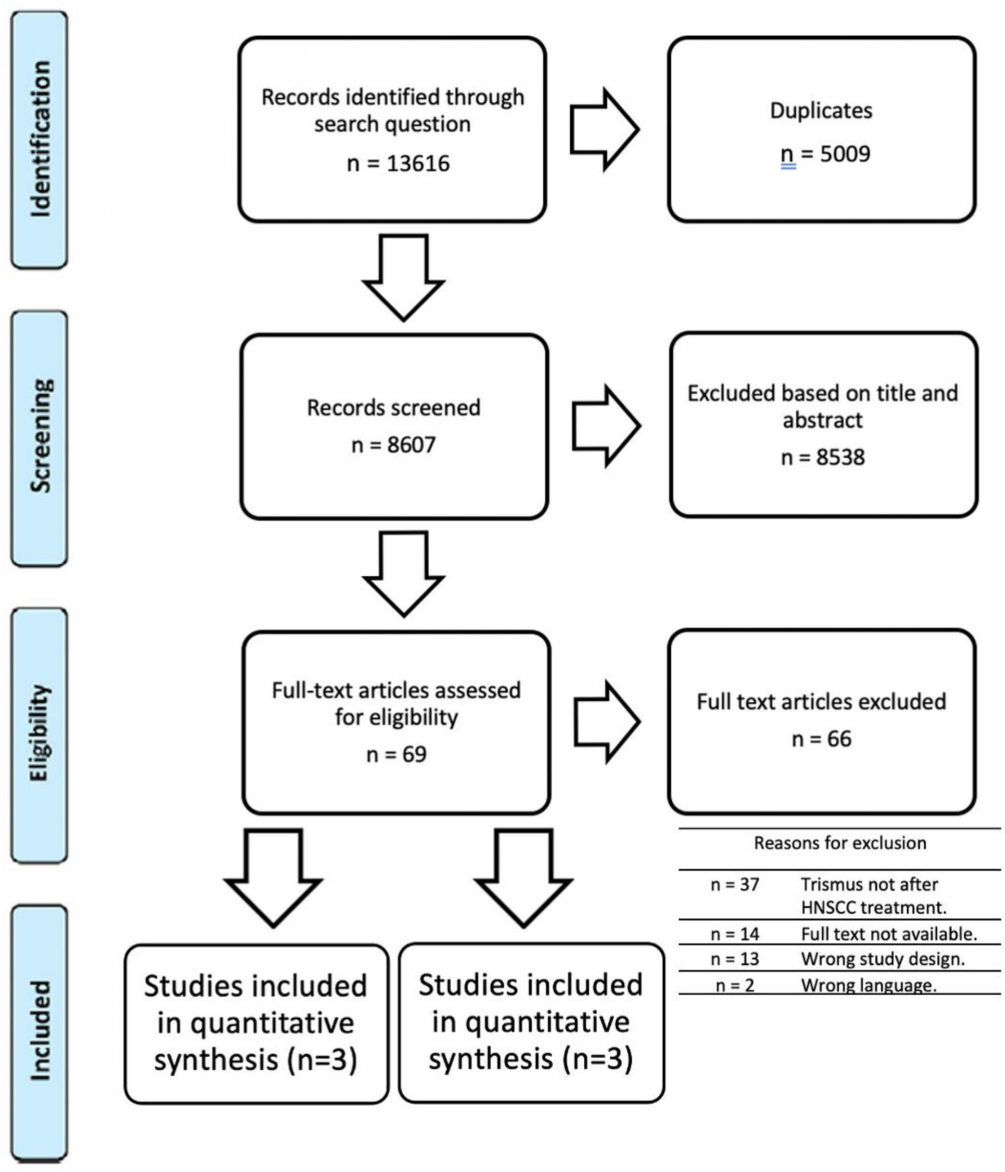
Prisma flow chart.

Of the 69 articles that were assessed for eligibility, 37 were excluded as the population did not consist out of former HNSCC cases. Thirteen articles were assessed as a wrong study design such as: literature reviews [24, 25], studies without surgical trismus release [11, 26–29], cohorts with simultaneous release of the mouth opening during the primary tumorectomy [30–32], a different cause for the limited MMO [33, 34], and an inadequate FU [35]. Furthermore, 14 articles were not available, 2 articles were excluded as they were written in a language apart from English, German, French, Spanish or Dutch. An overview of the selection and screening process is shown in Figure 1.

### Study Characteristics and Individual Results of the Included Papers

Bhrany et al. [20] described a mean gain in MMO of 21.8 mm in this population of 11 cases, who all underwent a CN, without MT or FFR. A mean gain at the end of FU of 8.9 ± 7.0 mm was the outcome of De Pablo et al. [21] analyzing the role of a FFR with (*n* = 17) or without (*n* = 11) a CN. Lastly, Mardini et al. [22] reached a gain in MMO between 1 and 20 mm using a technique combining CN, FFR and MT. Two studies included in the analysis had an intermediate risk of bias [20, 22] and one had a low risk of bias [21].

The demographic factors were described in Table 2. All three articles used a different subdivision for the tumor localization, so a detailed analysis of the localization was assumed too heterogeneous. Although, the buccal mucosa can be considered as the most common localization based on the finding that 25 out of a total of 46 cases were described as located in the buccal mucosa [20–22].

**Table 2:** Demographics of the included cases.

	**Bhrany et al. [20]**	**de Pablo et al. [21]**	**Mardini et al. [22]**
*n*	11	28	7
Male/female ratio	NS	26/2	6/1
Tumor localization	5 tonsil 6 palate	• 19 buccal mucosa • 3 alveolar ridge • 2 retromolar trigonum • 2 lip • 2 soft palate • 1 tongue	• 4 buccal mucosa • 2 buccal mucosa and maxillary bone 1 maxillary bone
Primary resection	5	28	7
Maxillectomy	3	15^a^	3^a^
Mandibulectomy	-	19^a^	-
Buccal mucosa resection	-	26^a^	6^a^
Tonsillectomy	2	-	-
Cheek through and through defect	-	2^a^	-
Free flaps harvested	2	28	7
Osteocutaneous	-	3	1
Fasciocutaneous	2	25	6
Radiotherapy (yes/no)	11	28	5
Chemotherapy (yes/no)	NS	18	NS
Time after primary treatment (m)	7–15	6–91	7–37
Mean FU after release (m)	12	38	31
Minimal FU (m)	12	12	7

a *combination of multiple defects described. n, number of cases; NS, not specified; m, months; FU, follow-up*.

### Results of Synthesis and Statistical Analysis

A significant increased gain in MMO was found if no primary surgery was executed (Nagelkerke *R2* = 0.350; *P* = 0.014^*^). No significant advantage was detected regarding the type of free flap during primary treatment (*R2* = 0.083; *P* = 0.226) or the administration of radiotherapy (*R2* = 0.089; *P* = 0.327).

Table 3 illustrates the mean increase in MMO at the different time points for each of the three methods.

**Table 3:** The mean maximal mouth opening and standard deviation (SD) for three surgical techniques and time points: preoperative, perioperative and at the end of follow-up.

**Surgical technique**	***n***	**Time (m)**		
		**Preoperative**	**Perioperative**	**End of follow-up**
**Myotomy**	7	4.14 ± 5.18	32.43 ± 3.36	18.57 ± 8.79
**Coronoidectomy**	35	8.94 ± 6.75	39.43 ± 5.82	23.69 ± 11.51
**Free flap release**	35	6.37 ± 4.91	36.80 ± 5.41	16.46 ± 7.01

*m, months*.

**Figure 2:**
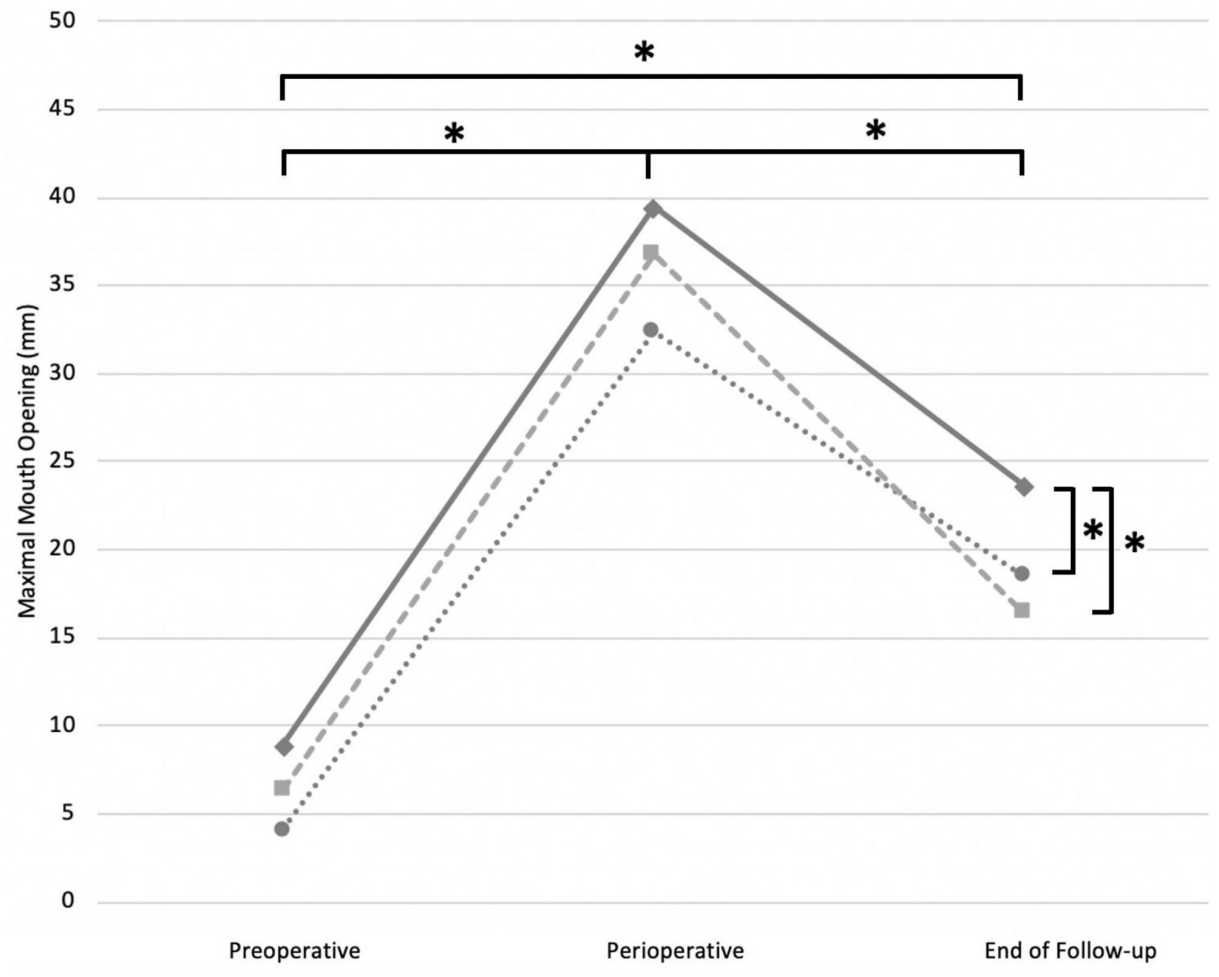
Estimated marginal means preoperative, perioperative and at the end of FU described for each surgical intervention strategy, when performed alone or in combination with one of the two strategies. Solid line, CN; dotted line, MT; striped line, FFR. *, *P*-value < 0.05 was set as statistically significant.

A main effect of surgical procedure group was found overall significant between the described release methods (*F* = 11.16; *P* < 0.01^*^). The MMO in the CN group (mean 24.02 ± 15.02 mm) was significantly (*P* < 0.01^*^) improved compared with the MT group (mean 18.38 ± 13.22 mm), and the FFR group (mean 19.88 ± 13.97 mm). No significant difference was observed between MT and FFR groups (*P* = 1.00) (Figure 2).

A significant effect of time was also noted between the three time points (*F* = 195.01; *P* < 0.01^*^). The perioperative MMO (mean 37.60 ± 5.78 mm) was the largest (*P* < 0.01^*^) compared with preoperative (mean 7.34 ± 5.98 mm) and postoperative MMO (mean 19.94 ± 9.98 mm). A significant improvement of the postoperative compared with the preoperative MMO was also noted (*P* < 0.01^*^) (Figure 2).

There was no interaction between time points and release method groups, indicating that the three surgical interventions exposed a similar evolution of the MMO over time (*F* = 1.492; *P* = 0.206).

## Discussion

Despite advances in the surgical techniques of head and neck cancer treatment, adequate long-term functional results are not always achieved. Limitation of mouth opening is one of the major factors leading to functional impairment. Secondary trismus release can be achieved by a variety of techniques. This systematic review identified three possible surgical techniques: FFR, CN and MT. A subsequent statistical analysis in a total of 46 patients identified the largest gain in CN.

A systematic review by Bouman et al. [36] described different therapeutic options for trismus release, although a majority of the included studies covered patients with OSF. Since the pathogenesis is different in OSF compared to HNSCC, we decided to exclude these patients from this analysis [37]. First of all, OSF is most frequently caused by betel nut chewing and is associated with superficial buccal scar tissue. On the other hand, deeper scar tissue is expected after extensive surgical reconstructions and radiotherapy for HNSCC. Furthermore, both of these treatments not only create scar tissue but also affect the availability of blood vessels and even perfusion in the head and neck area.

The main result of this statistical analysis is the significant gain in MMO in the group where a CN was effectuated compared with other methods of trismus release. Kumar et al. have published the beneficial effect of CN in Sawhney's type I-III temporomandibular joint ankyloses. A gain of 76% at least 1 year after surgery was shown in their population of 23 cases [38]. Similar benefit could hence be expected in trismus resulting from HNSCC treatment. It would be interesting to investigate specific variables affecting the MMO after HNSCC such as the role of coronoid size and hyperplasia.

Based on this analysis, scar tissue release with FFR was significantly less effective for MMO increase compared with the CN group. Comparison between these groups is however biased as preoperative MMO was lower in the population were a FFR was performed. These findings might suggest the difficulty of gaining an important quantity of MMO if the initial MMO is limited until just a few millimeters. Despite these noteworthy findings, no important conclusion can be made based on this small sample size regarding a FFR in trismus release.

No significant advantage of a myotomy was perceived in the analysis, nor in the individual studies. A myotomy is seen as one of the most accessible methods of trismus release, but none of the original research teams conducted a MT without a FFR or a CN. The overall consensus is that solely a MT is insufficient in releasing the MMO. One of the reasons for the latter is that the installed fibrous tissue after HNSCC is the major factor contributing to chronic trismus, especially for more severe trismus cases [39]. This was supported by the fact that all but one of the included articles described the simultaneous resection of the surrounding fibrous tissue. Furthermore, the reformation of fibrosis after a MT is to be expected with consequent recurrent trismus. Silberstein et al. identified the possible additional role of Botulinum toxin A in the MT procedure. According to this study, administration of Botulinum toxin A into a muscle immediately after MT might interfere with muscle healing, thus contributing to a more successful long-term result [40].

Subgroup analysis of the primary treatment revealed that a higher gain in MMO is to be expected after trismus release if no primary resection was performed, which can be attributed to fibrous scar tissue formation after primary surgery. Despite extensive reports on the role of radiotherapy as one of the main predictive factors for trismus, only little is known on the impact of the surgical resection [13, 14]. This is due to the impaired differentiation regarding the cause of the limited MMO between radiotherapy, surgery and an increased tumor staging [6, 13, 14]. No evidence was found for the lower reversibility of the MMO after trismus release due to radiotherapy or the type of FFR during primary treatment, which is most likely because of the low number of cases, respectively, with composite free flaps and without radiotherapy in this sample. Current scientific evidence suggests a lower trismus incidence is to be expected since the introduction of the intensity-modulated radiotherapy (IMRT) [14].

The loss of MMO between perioperative and the end of FU was noticed in all 46 cases, indicating the degree of trismus refractoriness that is to be expected. Immediate beginning of physical therapy and a mouth-exercising device [e.g., Therabite (Atos Medical, Malmö, Sweden) or Jaw Dynasplint (Dynasplint Systems, Severna Park, Maryland, USA)] might support the preservation of gained MMO. Nevertheless, it remains a matter of debate whether the perioperative measurement is significantly affected by induction and perioperative absence of pain limiting MMO.

A first limitation in this study is the limited number of eligible articles. The increasing disease-free survival due to new HNSCC treatment modalities explains the current shift toward a raising interest in the posttreatment quality of life and, thus, trismus. Therefore, the available number of articles regarding trismus after HNSCC treatment was considered disappointingly little. Secondly, the three described release methods were often combined, which hinders the differentiation between the used methods and their separate effect on the MMO. The third limitation is the multifactorial nature of this complication, despite addressing this with narrowing of the inclusion criteria to only HNSCC cases at least 6 months after oncologic treatment. Therefore, a higher sample size is needed for subgroup and multivariate analysis.

## Conclusion

Three methods were discovered for trismus release after HNSCC treatment: CN, MT and FFR. The currently available results support the low threshold for performing a CN in less severe limitation of the MMO. There is, despite the given results, a clear role for a FFR after scar tissue release for primary closure of the created defects, but the impact of a MT after scar tissue resection is still unclear. Further research is indispensable to reproduce the given studies on a larger homogeneous population to allow understanding of the surgical options in cases with a more severe objective and subjective limitation of the MMO.

## Data Availability Statement

Publicly available datasets were analyzed in this study. This data can be found here: doi: 10.1097/MLG.0b013e31812eee13; doi: 10.1002/jso.24806; doi: 10.1097/01.prs.0000221118.31863.c4.

## Author Contributions

All authors contributed in a different way regarding the conception and design of this review, acquisition of data *via* literature search, analysis and interpretation of data collected, drafting of the critical revision, and final approval of the manuscript.

## Conflict of Interest

The authors declare that the research was conducted in the absence of any commercial or financial relationships that could be construed as a potential conflict of interest.

## Publisher's Note

All claims expressed in this article are solely those of the authors and do not necessarily represent those of their affiliated organizations, or those of the publisher, the editors and the reviewers. Any product that may be evaluated in this article, or claim that may be made by its manufacturer, is not guaranteed or endorsed by the publisher.
