# Supplementary material for: The Effectiveness of Surgical Methods for Trismus Release at Least 6 Months After Head and Neck Cancer Treatment: Systematic Review
Source: Front Oral Health. 2022 Jan 21;2:810288. doi: 10.3389/froh.2021.810288 (PMC8814314; doi:10.3389/froh.2021.810288)
Supplement: Supplementary file 1 [file Data_Sheet_1.docx]

Search strategy.

| **Keywords** | **1: surgical interventions for trismus release** | **2: trismus or restricted mouth opening** |
| --- | --- | --- |
| PUBMED central | (“trismus release”[TIAB] OR “oral surgery”[MeSH] OR surgeon[TIAB] OR surgeons[TIAB] OR surgeries[TIAB] OR surgery[TIAB] OR surgical[TIAB] OR surgically[TIAB] OR surgicals[TIAB] OR “operative surgical procedures”[MeSH] OR operative-procedure*[TIAB] OR oropharyngectomie*[TIAB] OR pharynx-extirpation*[TIAB] OR head-and-neck-reconstruction*[TIAB] OR operative-reconstruction*[TIAB] OR “myotomy”[MeSH] OR myotomy[TIAB] OR myotomie*[TIAB] OR coronoidectomy[TIAB] OR coronoidectomie*[TIAB] OR “free tissue flaps”[MeSH] OR flap*[TIAB] OR graft*[TIAB] OR “mandibular reconstruction”[MeSH] OR mandibular-reconstruction*[TIAB]) | (“trismus”[MeSH] OR trismus[TIAB] OR “maximal interincisal opening”[TIAB] OR MMO[TIAB] OR MIO[TIAB] OR (opening[TIAB] AND ("mouth"[MeSH] OR "mouth"[TIAB])) OR “mouth opening”[TIAB] OR Lockjaw[TIAB] OR “Lock Jaw”[TIAB] OR Masseter-Muscle-Spasm*[TIAB] OR Masseter-Spasm*[TIAB]) |
| Embase | (((‘trismus release’ OR ‘coronoidectomy’ OR ‘coronoidectomie*’ OR ‘myotomie*’ OR ‘myotomy’ OR ‘operative procedure’ OR ‘mandibular reconstruction*’ OR ‘graft*’ OR ‘flap*’ OR ‘oropharyngectomy’ OR ‘pharynx extirpation’ OR ‘head neck reconstruction’ OR ‘operative reconstruction’ OR ‘surgeon’ OR ‘surgeons’ OR ‘surgeries’ OR ‘surgery’ OR ‘surgical’ OR ‘surgically’ OR ‘surgicals’):ti,ab,kw) OR ((‘coronoidectomy’ OR ‘myotomy’ OR ‘surgery’ OR ‘surgeon’ OR ‘ear nose throat surgery’ OR ‘head and neck surgery’ OR ‘reconstructive surgery’ OR ‘free tissue graft’ OR ‘free vascularized fibular graft’)/exp)) | (((‘trismus’ OR ‘lockjaw’ OR ‘lock jaw’ OR ‘masseter muscle spasm’ OR ‘masseter spasm’ OR ‘mouth opening’ OR ‘interincisal opening’ OR ‘MMO’ OR ‘MIO’):ti,ab,kw) OR (‘opening’:ti,ab,kw AND (‘mouth’/exp OR ‘mouth’:ti,ab,kw)) OR ((‘trismus’ OR ‘mouth opening’)/exp)) |
| Web of Science | TS=((“trismus release” OR “surgeon” OR “surgeons” OR “surgeries” OR “surgery” OR “surgical” OR “surgically” OR “surgicals” OR “head and neck reconstruction*” OR “operative procedure*” OR “oropharyngectomie*” OR “pharynx extirpation*” OR “myotomy” OR “myotomie*” OR “coronoidectomy” OR “coronoidectomie*” OR “flap*” OR “graft*”) | TS=(“trismus” OR “maximal interincisal opening” OR “MMO” OR “MIO” OR (“mouth” AND “opening”) OR “mouth opening” OR “Lockjaw” OR “Lock Jaw”)) |
| Cochrane database | #1 : ([mh “surgery, oral”] OR [mh “surgical procedures, operative”] OR [mh “myotomy”] OR [mh “free tissue flaps”] OR [mh “mandibular reconstruction”])  #2 : (((trismus NEXT release) OR surgeon OR surgeons OR surgeries OR surgery OR surgical OR surgically OR surgicals OR (operative NEXT procedure*) OR myotomy OR myotomie* OR coronoidectomy OR coronoidectomie* OR flap* OR graft* OR (mandibular NEXT reconstructi*)):ti,ab,kw)  #3 : #1 OR #2 | #4 : ([mh “trismus”])  #5 : ((trismus OR (maximal NEXT interincisal NEXT opening) OR MMO OR MIO OR (opening NEXT of NEXT the NEXT mouth) OR (mouth NEXT opening) OR Lockjaw OR Lock NEXT Jaw):ti,ab,kw)  #6 : #5 OR #6 |
| OpenGrey | (“trismus release” OR “oral surgery” OR “surgeon*” OR “surgeries” OR “surgery” OR “surgical*” OR “oropharyngectomie*” OR “pharynx-extirpation*” OR “head and neck reconstruction*” OR “myotomy”OR “myotomie*” OR “coronoidectomy” OR “coronoidectomie*” OR “free tissue flaps” OR “mandibular reconstruction”) | (“trismus” OR “maximal interincisal opening” OR “MMO” OR “MIO” OR (“opening” AND "mouth") OR “mouth opening” OR “Lockjaw” OR “Lock Jaw” OR “Masseter-Muscle-Spasm*” OR “Masseter-Spasm*”) |
